# Archived DNA reveals fisheries and climate induced collapse of a major fishery

**DOI:** 10.1038/srep15395

**Published:** 2015-10-22

**Authors:** Sara Bonanomi, Loïc Pellissier, Nina Overgaard Therkildsen, Rasmus Berg Hedeholm, Anja Retzel, Dorte Meldrup, Steffen Malskær Olsen, Anders Nielsen, Christophe Pampoulie, Jakob Hemmer-Hansen, Mary Susanne Wisz, Peter Grønkjær, Einar Eg Nielsen

**Affiliations:** 1 Section for Marine Living Resources, National Institute of Aquatic Resources, Technical University of Denmark, Vejlsøvej 39, 8600 Silkeborg, Denmark; 2 Greenland Climate Research Centre, Greenland Institute of Natural Resources, Kivioq 2, PO Box 570, 3900, Nuuk, Greenland; 3 Landscape Ecology, Institute of Terrestrial Ecosystems, ETH Zürich, Zürich, Switzerland; 4 Swiss Federal Research Institute WSL, 8903, Birmensdorf, Switzerland; 5 Hopkins Marine Station, Department of Biology, Stanford University, 120 Oceanview Blvd, Pacific Grove, CA 93950, USA; 6 Greenland Institute of Natural Resources, Kivioq 2, PO Box 570, 3900, Nuuk, Greenland; 7 Research and Development, Danish Meteorological Institute, Lyngbyvej 100, 2100 Copenhagen, Denmark; 8 Section for Marine Living Resources, Technical University of Denmark, Jægersborg Allé 1, 2920 Charlottenlund, Denmark; 9 Marine Research Institute, Reykjavık, Iceland; 10 Section for Ecosystem Based Marine Management National Institute of Aquatic Resources Technical University of Denmark Jægersborg, Allé 1, 2920 Charlottenlund, Denmark; 11 Department of Bioscience - Aquatic Biology, Ole Worms Allé 1, 8000 Aarhus, Denmark

## Abstract

Fishing and climate change impact the demography of marine fishes, but it is generally ignored that many species are made up of genetically distinct locally adapted populations that may show idiosyncratic responses to environmental and anthropogenic pressures. Here, we track 80 years of Atlantic cod (*Gadus morhua*) population dynamics in West Greenland using DNA from archived otoliths in combination with fish population and niche based modeling. We document how the interacting effects of climate change and high fishing pressure lead to dramatic spatiotemporal changes in the proportions and abundance of different genetic populations, and eventually drove the cod fishery to a collapse in the early 1970s. Our results highlight the relevance of fisheries management at the level of genetic populations under future scenarios of climate change.

Global change impacts the abundance and distribution of biodiversity in the world´s oceans, which in turn affects the services provided by marine ecosystems^1^,^2^. Marine fisheries are an invaluable resource supporting human welfare worldwide^3^. Historically, dramatic changes in both the abundance and distribution of many important fish stocks have been observed^4^,^5^,^6^,^7^,^8^. However, the relative impact of fishing and climate on stock dynamics is still under debate^9^, as few studies have been able to assess the integrated responses of fish stocks to these pressures.

Many marine fish species are made up of genetically distinct populations that often do not match traditional fisheries management units, “stocks”^10^,^11^. Stocks are defined geographically rather than biologically, and can potentially lead to overexploitation of more vulnerable populations, in a fishery consisting of a mixture of different fish populations^12^, a so-called “mixed-stock fishery”. Genetic populations may exhibit unique adaptations and tolerances to specific environments^13^, and might display distinct responses to fisheries and climate change^14^,^15^. Disentangling how different populations have responded to intense fishing pressure and climate variability could hold key answers for understanding historical fish distribution and abundance patterns and for improved future management of marine fish resources.

Here, we used a large archive of fish earstones (otoliths) to study the population dynamics of Atlantic cod (*Gadus morhua*) during the historical commercial fishery in West Greenland, which displayed a dramatic collapse similar to a number of other cod fisheries^16^,^17^,^18^,^19^. From peak landings that ranged between 400 K and 500 K tons in the 1960’s, the catch dropped dramatically through the 1970’s to a complete collapse in the early 1990s^20^,^21^ (see Fig. 1), and have since remained low^21^. Traditionally, biomass increases and large catches were partly ascribed to a generally warmer climate and the influx of cod eggs and larvae from Iceland^22^,^23^, but no clear explanation of the population dynamics has emerged. Recent work has shown that at least four genetically distinct cod populations occur along the coasts of Greenland^24^, presenting an opportunity to examine fishery dynamics within distinct populations.

Over the last century, otoliths have been collected for age and growth determination, and now also provide an opportunity for recovering historical DNA^25^,^26^. In this study, DNA was extracted and analyzed from 872 cod otoliths and a panel of 81 gene-associated Single Nucleotide Polymorphisms (SNPs)^27^ was applied to allow the assignment of each cod individual to its genetic populations of origin among those described within Greenlandic waters^24^: West Greenland offshore, West Greenland inshore, Iceland offshore (also known as East Greenland/Iceland offshore population^28^) and Iceland inshore, (population names re-adapted from previous study^24^). We generated a probability distribution of the contribution of the different genetic populations to the total catch over time and related temporal variation in catch composition within fisheries management areas to historical sea surface temperatures using ecological niche modeling^29^.

Genetic assignment of archived otoliths revealed different spatiotemporal contributions of populations to the historical commercial fishery (Fig. 2a). Catches during the 1930s consisted almost exclusively of cod from the local West Greenland offshore population (98.8%). The contribution from this population decreased dramatically during the peak fishing in the 1950s (46.5%) and 1960s (34%), but in the 1970s and 1980s this population had almost disappeared from the catch in the southwest Greenland fishing areas (NAFO divisions 1D to1 F, 1.1%, Fig. 2a), but still appeared to be present in the northern areas. In contrast, the contribution from the Icelandic offshore population with spawning areas offshore Iceland and offshore East Greenland^23^ increased from the start of the major fishing boom in the 1950s (29.1%) to constitute the vast majority of the catch during and immediately following the stock collapse around the 1970s (91.6%).

The collapse of the West Greenland offshore population was predicted from the fishing intensity and productivity estimates of the two main populations. The average spawning stock biomass needed to produce one recruit (3 years old) was 5.56 kg for the West Greenland offshore cod compared to 1.06 kg for the Iceland offshore cod, which corresponds to equilibrium fishing mortalities (F_eq_) of 0.14 and 0.82 respectively^30^ (see details in Methods). These estimates are sensitive to errors in the estimation of spawning stock biomass and recruitment, where overestimation of spawning stock biomass and underestimation of recruitment will lead to inflated F_eq_. A sensitivity analysis showed that the West-Greenland spawning stock biomass during 1955–1972 would have been overestimated by a factor of five to allow for a F_eq_ corresponding to the Icelandic F_eq_. Within all reasonable scenarios of errors, the West-Greenlandic F_eq_ is considerably lower than the Icelandic F_eq_ (see details in Supplementary Information). Accordingly, historically observed intermediate fishing intensities (between the two F_eq_ estimates)^30^ would sustain the Icelandic offshore cod population while leading to the collapse of the West Greenland offshore cod population. This pattern was observed during 1950–1968 when the biomass of the West Greenland offshore population plummeted while the biomass of the Iceland offshore population remained stable (Fig. 2b). Following the crash and the post-1990 absence of commercial landing in the northern and central NAFO divisions^31^, the local West Greenland offshore population has rebounded in terms of contribution to the mixed stock (Fig. 2a). This increase has primarily been seen in the central areas (division 1D; Fig. 2a). The contribution from the Icelandic offshore population has generally decreased but remained very high in the southern NAFO divisions. Throughout the period, West Greenland inshore and Icelandic inshore populations were present, but never constituted a major part of the catch.

We related the shift of the proportion of the two major stock components, (i.e. West Greenland offshore relative to the Iceland offshore populations) to the historical climatic suitability for cod along West Greenland area (see details in Methods). We estimated yearly cod climate suitability using species distribution models, hindcasted to 1948–2011 hydrographic conditions. Historical climate suitability was estimated both at the species level (mean model accuracy measured with Area Under the Curve (AUC) = 0.83), as well as for distinct populations assuming population-level genetic adaptations (AUC = 0.78). For each year and each 50 × 50 km pixel, we computed the least-cost path distance along the coast through climatically suitable conditions to the southern tip of Greenland (presumed entry point of Icelandic offshore cod into West Greenlandic waters). We found that the spatiotemporal shift in the proportion of the Iceland offshore population relative to that from offshore West Greenland was better explained by a population level climatic niche assuming distinct genetic adaptation for the population (general linear model with a binomial distribution, coefficient of determination R^2^ = 0.41, slope = −0.04, Wald-z test p = 0.001; Fig. 3a), than considering a general climatic requirement for the stock as a whole (R^2^ = 0.30, slope = −0.05, p = 0.03) or static geographic distance (R^2^ = 0.29, slope = −0.06, p = 0.03, Fig. 3b). Moreover, the proportion of Icelandic cod catches was best explained by the least cost path distance to spawning areas via oceanographic routes with suitable conditions (e.g. surface temperatures) in contrast to the shortest geographic route to spawning areas (Fig. 3a,b). We also observed that the West Greenland offshore population occured predominantly in colder sea surface temperatures, while the Iceland offshore population occupied a relatively broader range of temperature conditions including warmer temperatures (see Supplementary Figure S2). The climatic niche modelling documented an increased suitability of the West Greenland area for the Icelandic offshore population during the warm periods, facilitating the distribution at higher latitude areas. A regime shift detection analysis applied to the habitat suitability data showed a significant decrease concurrent with the collapse of the fishery during the cold period beginning in 1970s (see Methods). This low suitability regime persisted until 1997 only interrupted by a short improvement in the period 1977–1982 (Fig. 3c).

This study demonstrates the value of using DNA from archived fish in order to identify vital processes at the population level for understanding the impact of exploitation and climate change on the historical distribution of marine fish. First, we showed that the high and indiscriminate fishing pressure resulted in a collapse of the local, cold-adapted, West Greenland offshore population in the 1970s. Concurrent with this collapse, the colder conditions prevented the Icelandic cod population from increasing in abundance, and the flourishing West Greenland cod fishery vanished.

Our results suggest that tracking the genetic origin of harvested fish could support spatially differentiated management plans and help avoid a disproportional impact on the most vulnerable populations. This will have important consequences for fisheries management in West Greenland and require that separate quotas are set for the different biological populations. However, we expect that maintenance of this biocomplexity will lead to more stable ecosystem services in the future due to the portfolio effect^13^, whereby today’s strained cod populations may thrive under future conditions. A number of previous studies have shown that species are not equal in the face of climate change, with some geographic ranges expanding while others contract^22^. Our results suggest that within species, different, locally-adapted genetic pools may win or lose disproportionally under climate change. If not counteracted through population based fisheries management, exploitation may lead to the collapse of populations needed to adapt to future environmental conditions.

## Methods

### Sampling

Archived tissue samples (otoliths and scales) of Atlantic cod (*Gadus morhua*) were originally collected off west Greenland through a combination of commercial fishing, during annual surveys (with R/V Paamiut) and as part of the cod tagging program conducted by the Greenland Institute of Natural Resources in Nuuk, Greenland. 872 samples were collected between late June and January in: 1932, 1952, 1962, 1977, 1980, 1989, 2000, 2008 and 2012 (see Supplementary Table S1 and Supplementary Table S2). Sampling was stratified according to the Northwest Atlantic Fisheries Organization Convention Area in West Greenland (NAFO Subarea 1; from 1A to 1F divisions). Further details about seasonal composition of sampling are outlined in Supplementary Information.

### SNPs selection and genotyping

81 SNPs (Single Nucleotide Polymorphisms) were selected as the most informative for population assignment out of a panel of 935 SNPs that was recently screened in spawning population samples collected throughout Greenlandic and Icelandic waters^24^. The reduced panel was selected to achieve the minimum assays with maximum power and the selected loci showed the highest *F*_*CT*_(differentiation between groups) in pairwise comparisons of the four distinct spawning groups identified. For this study, a total of 867 cod individuals were genotyped for the 81 SNP panel using the Fluidigm 96.96 Dynamic Arrays system (BioMark HD System) following the instructions from the manufacturer and standard methods^32^.

### Individual assignment tests

Two different genetic assignment approaches were used to estimate the historical contribution of different cod populations to the fished stock. Individual assignment test was first conducted with the program GENECLASS2^33^. These tests were based on the Bayesian probability approach^34^ and a Monte-Carlo resampling method for probability computation^35^ with 10000 simulated individuals (α = 0.01) to evaluate the probability that a certain multilocus genotype originated from one of the four baseline populations previously identified^24^ (populations names were changed according to defined spawning grounds^28^). Furthermore, Discriminant Analysis of Principal Components (DAPC^36^) implemented in the adegenet R package was also employed. In this multivariate framework, the mixed stock individuals were assigned with the predict.dapc() function that projects multilocus genotypes onto discriminant functions (i.e. synthetic variables that maximize differences between and minimize differences within *a priori* defined reference groups), thereby deriving posterior membership probabilities to each reference group (spawning population) for each individual. In both approaches, the baseline genetic signature (allele frequencies) of each spawning population was defined based on individual samples collected at the spawning time for a previous study^24^, further details are outlined in Supplementary Information. Only mixed stock individuals that could be assigned with >0.90 probability to a particular population with both methods were included in the following analysis. Individuals assigned with lower probability or with inconsistent results for the two methods were discarded. However, individual assignment test with GENECLASS2 was generally highly consistent with that of DAPC, and 85% of individuals were assigned to one of the genetic populations inhabiting Greenlandic waters using the above criteria. Out of 872 individuals analyzed, 103 were discarded.

### Probability distribution of total catch composition over time

At each observation time *t*_*i*_, *i* = 1 … *n*, the total number of investigated fish *N*_*i*_ was categorized into four genetically distinguished spawning groups: West Greenland offshore (1), West Greenland inshore (2), Iceland offshore (3), and Iceland inshore (4) (Supplementary Figure S1). These observations are naturally described by a multinomial distribution with sample size *N*_*i*_. To describe the development of the stock proportions over time it is assumed that the probability vector: *p*_*t*_ = (*p*_*1*_, *p*_2_, *p*_3_, *p*_4_) follows an unobserved process. The process is set up such that the sum of *p*_*t*_is 1 and such that the elements of *p*_*t*_each are between 0 and 1. The following transformation defines the process: let *α*_*t*_ = (*α*_*t*1_, *α*_*t*2_, *α*_*t*3_) follow a three dimensional random walk, such that: *α*_*t*_ = *α*_*t*−1_ *+* *ε*_*t*_, where *ε*_*t*_ *~* *N* (0, σ^2^I_3×3_). Then, we defined 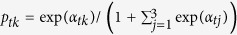, for *k* = 1 … 3, and 
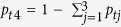
.

**Figure 1:**
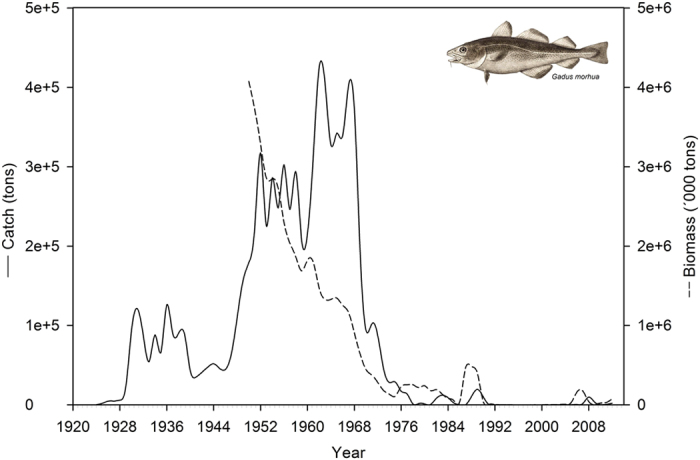
Historical Atlantic cod (*Gadus morhua*) biomass (dotted line) and commercial catch (solid line) in West Greenland (readapted after^20^,^21^). This figure has been drawn by S.B. using SigmaPlot 12 software.

The catch probability distributions were multiplied by the total biomass of fish age 3 years and older (*30*) in order to estimate the biomass contribution of each population during 1950–2012 (Fig. 1).

### Equilibrium fishing mortalities

The equilibrium fishing mortality (F_eq_) is the fishing mortality that balances mortality and reproduction, ensuring that enough fish survive to reproduce to exactly replenish them via recruitment^37^. One approach to determine F_eq_ is to calculate the spawning biomass needed to produce one new recruit from time series of spawning stock biomasses and recruitment, and subsequently use an exponential decay function (1) to search for the equilibrium fishing mortality (F_eq_) that would lead to this equilibrium biomass. Actual fishing mortalities can then be evaluated against F_eq_ to reveal whether a current or historical fishing pattern was sustainable. This analysis was performed for the West Greenland offshore and Icelandic offshore populations. The recruitment per spawning stock biomass (R/SSB) index of fish stock productivity was obtained from^30^ for both the West Greenlandic offshore and Icelandic offshore populations. For the period 1924 to 1973 the average SSB needed to produce 1 new recruit, and hence ensure replacement of the adult population by new recruits, was 5.56 g for West Greenland offshore cod compared to 1.06 kg for the Icelandic offshore cod during 1955–2002. The period 1924–1973 was chosen because this represents a period when recruitment of cod in west Greenland was driven by the local West Greenlandic offshore population.

We used the exponential decay function to search for the equilibrium fishing mortality (F_eq_) for the age groups 5 to 12 years that would lead to an average spawning biomass of 5.56 kg and 1.06 kg. Age group specific fishing mortality and natural mortality, weight-at-age and maturity-at-age for the West Greenlandic offshore population was also obtained from^30^ whereas these data were obtained from^38^ for the Icelandic offshore population. We used the exponential decay function (1): N_t+1_ = N_t_ * exp^−(F+M)^, where N_t_ is the number of fish alive at time t, F is the instantaneous fishing mortality and M is the instantaneous natural mortality, to calculate the numbers of fish alive at in each age group (3 to 12 years) given the F and M. These numbers were then multiplied by the weight-at-age and maturity-at-age to yield the spawning stock biomass, and, subsequently scaled to one initial recruit, i.e. the stock size at age 3 was set to one fish (Supplementary Table S3 and Supplementary Table S4). The calculated average F_eq_ was scaled to the age-specific fishing pattern obtained from a virtual population analysis (VPA) for the period 1961–1963. The low productivity of Greenlandic cod only allowed for an equilibrium F_(5–12)_ of 0.14 (Supplementary Table S3). A similar calculation for the Icelandic cod stock using SSB/R of 1.06 kg corresponded to an equilibrium F_(5–12)_ of 0.82 (Supplementary Table S4). A fishing mortality that exceeds F_eq_ will lead to reduced SSB and subsequently reduced R until the collapse of the stock. Consequently, a fishing mortality between 0.14 and 0.82 that would sustain the Icelandic offshore cod population would lead to the collapse of the West Greenland offshore cod population. It should be noted that the equilibrium fishing mortality for the Icelandic offshore cod is calculated for the total cod population, i.e. including the individuals residing in Icelandic waters. Hence, the actual fishing pressure of this population will be an abundance weighted mean of the fishing pressure in Icelandic and West-Greenlandic waters. To test the impact of changes in growth and maturity trajectories, and errors in the estimation of recruitment and spawning stock biomass on the equilibrium fishing mortalities we performed a sensitivity analyses outline in Supplementary Information.

### Habitat suitability modeling

The Greenland Institute of Natural Resources at Nuuk conducts scientific trawling primarily for shrimp and halibut each year, and also collects information on all other fish species (approximately 200 species, total, including cod). The surveys (with R/V Paamiut) are designed and stratified to provide core information relevant for stock assessment purposes. We modeled habitat suitability by combining the data from all four cod spawning populations, and at single population level (i.e. only including records identified as belonging to the Iceland offshore population) and projected/predicted these models to annual hydrodynamical scenarios (i.e. one from each year). For the mixed stock (all spawning populations combined), we modeled presence and absence of cod occurrences in the Paamiut scientific trawls. These data included n = 1263 presence and n = 1708 absence records. To compute the habitat suitability model at the population level, we modeled presences of Iceland offshore fish identified from genotyping of fish caught in the Paamiut trawls from 2000 to 2011, and absences were drawn from the absences recorded from the Paamiut surveys during the same period (n = 357 presence and 1708 absence records).

We used an ensemble forecasting approach that combined predictions of four modeling techniques including generalized linear models (GLM), generalized additive model (GAM), generalized boosting methods (GBM) and random forest (RF) as applied in^39^. We used a binomial linear model and down-weighted the absence records to match the number of presences^40^. For each modeling technique, 10 repetitions were performed using random sets of 80% of the initial occurrences to calibrate the model. The remaining 20% were used for the evaluation of the model using the Area Under the Curve^41^. The final model was then projected to past and future hydrographical scenarios (1948–2011), at 1 year-intervals averaging the four modeling techniques. Calculations were performed in R^42^.

**Figure 3:**
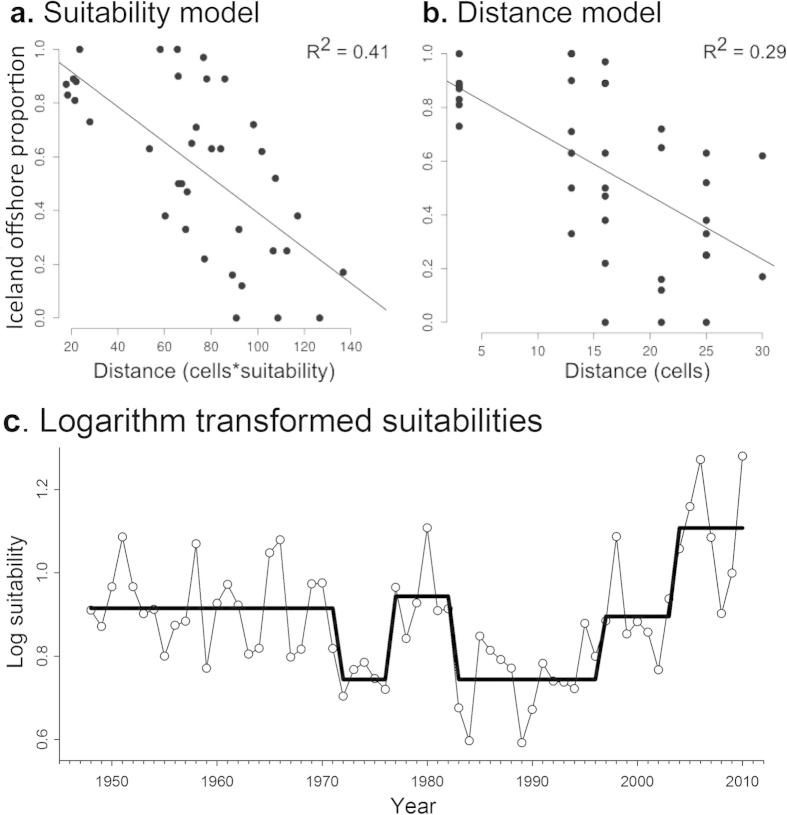
Climatic suitability for the Iceland offshore population linked to cod population structure in NAFO divisions. (**a**) Proportion of cod from the Iceland offshore population in a given NAFO division in relation to of least-cost path distance to nearest spawning areas weighted by suitability. (**b**) Proportion of cod from the Iceland offshore population as a function of the shortest sea distance to nearest spawning area. (**c**) Yearly averaged logarithm transformed random forest generated habitat suitability for Icelandic offshore cod along West-Greenland. Solid line is mean value from a regime shift detection analysis^40^ (cut-off: 5 years, significance level 0.1).

A regime shift detection analysis (Fig. 3c) based on logarithm-transformed average habitat suitability (based on the random forest algorithm) for Icelandic offshore cod was performed to reveal periods of differing habitat suitability^43^. Further details about hydrodynamical modeling data and evaluation of population level suitability with dispersal are reported in Supplementary Information.

## Additional Information

**How to cite this article**: Bonanomi, S. *et al.* Archived DNA reveals fisheries and climate induced collapse of a major fishery. *Sci. Rep.* **5**, 15395; doi: 10.1038/srep15395 (2015).

## Supplementary Material

Supplementary Information

## Figures and Tables

**Figure 2:**
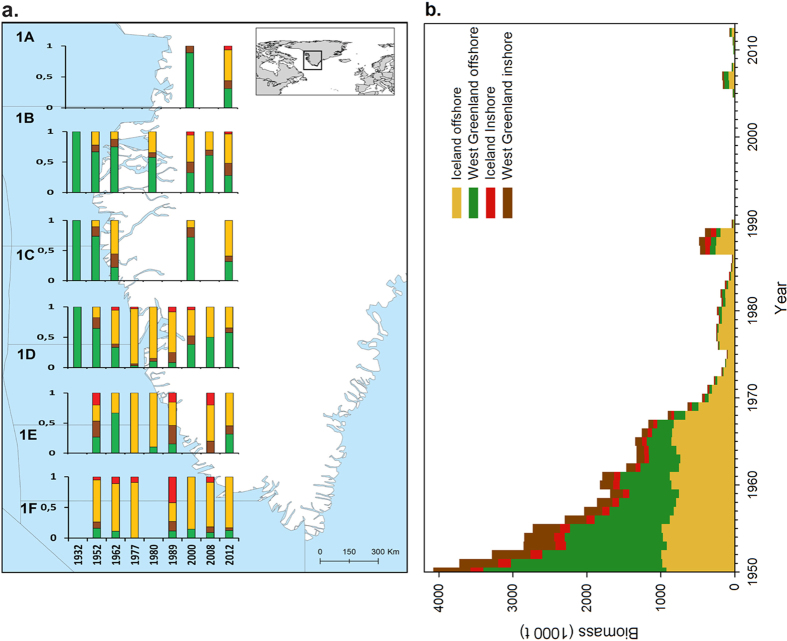
(**a**) Spatiotemporal development in the proportions of different Atlantic cod (*Gadus morhua*) populations in the historical West Greenland fishery (NAFO divisions from 1A to 1F): West Greenland offshore (green), West Greenland inshore (brown), Iceland offshore (dark yellow), Iceland inshore (red).The Greenland map and the small-scale world map have been drawn by S.B. using ArcGIS and R software respectively. (**b**) Estimated stock biomass composition of cod along West Greenland 1950–2012 (NAFO divisions 1A–1F). Biomass is estimated based on catch proportions (Supplementary Figure 1) and the biomass of 3+ years old cod in the stock.
