# Supplementary material for: Archived DNA reveals fisheries and climate induced collapse of a major fishery
Source: Sci Rep. 2015 Oct 22;5:15395. doi: 10.1038/srep15395 (PMC4614879; doi:10.1038/srep15395)
Supplement: Supplementary Information [file srep15395-s1.pdf]

SUPPLEMENTARY INFORMATION

Archived DNA reveals fisheries and climate induced collapse of a major fishery

Sara Bonanomi, [1, 2,] *† Loïc Pellissier, [3,] † Nina Overgaard Therkildsen, [2, 4] Rasmus Berg Hedeholm, [2, 5] Anja Retzel, [2, 5] Dorte Meldrup, [1] Steffen Malskær Olsen, [6] Anders Nielsen, [7] Christophe Pampoulie, [8] Jakob Hemmer-Hansen, [1] Mary Susanne Wisz, [9,] ‡ Peter Grønkjær, [2,10,] ‡ Einar Eg Nielsen [1, 2,] ‡

1 Section for Marine Living Resources, National Institute of Aquatic Resources, Technical University of Denmark, Vejlsøvej 39, 8600 Silkeborg, Denmark

2 Greenland Climate Research Centre, Greenland Institute of Natural Resources, Kivioq 2, PO Box 570, 3900, Nuuk, Greenland

3 Department of Biology, Unit of Ecology & Evolution, Chemin du Musée 10, 1700 Fribourg, Switzerland

4 Hopkins Marine Station, Department of Biology, Stanford University, 120 Oceanview Blvd, Pacific Grove, CA 93950, USA

5 Greenland Institute of Natural Resources, Kivioq 2, PO Box 570, 3900, Nuuk, Greenland

6 Danish Meteorological Institute, Lyngbyvej 100, 2100 Copenhagen, Denmark

7 Section for Marine Living Resources, Technical University of Denmark, Jægersborg Allé 1, 2920 Charlottenlund, Denmark

8 Marine Research Institute, Reykjavık, Iceland

9 Department of Ecology and Environment, DHI, Agern Allé 5, 2960 Hørsholm, Denmark

10 Department of Bioscience - Aquatic Biology, Ole Worms Allé 1, 8000 Aarhus, Denmark

* Email: sara.bonanomi@an.ismar.cnr.it, Tel: +39 071 2078830

Section Overview:

Section S1 – Sampling – Seasonal composition

Section S2 – Individual assignment tests – Temporal stability

Section S3 – Equilibrium fishing mortalities - Sensitivity analyses

Section S4 – Habitat suitability modeling - Hydrodynamical model data

Section S4 – Habitat suitability modeling - Evaluating population level suitability with dispersal

Section S5 – Supplementary Figure S1 to S5

Section S6 – Supplementary Tables S1 to S4

Section S1 – Sampling – Seasonal composition

The majority of samples analyzed (85%) were collected during summer months from June to September. This is consistent across NAFO divisions where "summer" samples constitute a minimum of 69% (mean 89%). Only for two years (1962 and 1980), samples collected in October/November constitute a major part of the total sampling (58% and 69% respectively). These differences in sampling time could potentially (but not necessarily) have an effect on population contributions for specific areas for those two years as illustrated in Figure 2a. However, given the general homogeneity in sampling time across NAFO divisions and years, it is highly unlikely that these specific differences in sampling time will have any effect on the larger picture (Figure 2b), with respect to the overall contribution to the fishery by different populations over time. Furthermore, for a few NAFO divisions/years the number of available samples was low, however, our conclusions are not based on individual samples but on the overall temporal trends as revealed by the probability distribution of total catch composition over time and the suitability modeling.

Section S2 – Individual assignment tests – Temporal stability

With both assignment approaches, the baseline genetic signature (allele frequencies) of each spawning population was defined based on individual samples collected at the spawning time for a previous study [1]. For three of the four populations, the reference dataset included samples of 25-59 individuals collected four-six decades apart. There was consistently less genetic differentiation between samples collected over time within the same reference location (Fst=<0.029) than there was between the reference populations (Fst >= 0.044). Assignment tests using only the contemporary samples as the reference also assigned 81-98% of the historical reference individuals to their correct population, indicating a high level of temporal stability in the genetic signatures for each population over the study period.

Section S3 – Equilibrium fishing mortalities - Sensitivity analyses

We performed a sensitivity analysis to examine the robustness of the equilibrium fishing mortality to changes in weight-at-age and maturity-at-age in the West Greenlandic offshore cod population, by increasing the weight and the proportion of mature fish at each age stepwise by 10% to 50% above the starting values and calculating the resulting Feq as above. The change in weight-at-age had the largest impact on Feq, but in none of the scenarios did Feq increase beyond the F observed during the collapse of the West Greenlandic offshore cod population (Supplementary Figure S3). Hence, increased growth and earlier maturity could not counter-balance the increasing fishing pressure during the 1950s and 1960s.

Because the equilibrium fishing mortalities were calculated from the spawning biomass and recruitment time-series, these numbers were influenced by errors in the estimation of these two quantities. We therefore ran matrix-of-error scenarios to evaluate the potential impact of errors on Feq. Using the average West-Greenlandic spawning stock biomass and recruitment from 1955-1972 [2], we estimated the Feq resulting from a matrix ranging from a ratio of 0.2 to 1.6 between true and estimated spawning stock biomass, and 0.4 – 2 between true and estimated recruitment (see Supplementary Figure S4). A ratio of 0.2 corresponds to a factor five overestimation, whereas a ration of two corresponds to a factor two underestimation of either spawning stock biomass or recruitment. This range of scenarios also covers the situation during the period of collapse of the West-Greenlandic population (1950-1968) where the proportion of West-Greenlandic cod contributed on average 38% percent of the total biomass. It's important to note, however, that the proportion of West-Greenlandic cod in the spawning stock biomass is likely to be an underestimate, as the Icelandic Offshore fish migrate back to East Greenland and Iceland at maturation. Only at extreme overestimations of spawning stock biomass (0.25 or factor 4) and underestimation of recruitment (half of true recruitment) did the West-Greenlandic Feq approach the Icelandic Feq (0.84). According to previous work [3], the fishing mortality rose above 0.5 around 1960. In order to sustain this fishing pressure, the spawning stock biomass per recruit should have been as little as 1.5 kg, which at the same recruitment level would have required that the true spawning stock was overestimated by a factor of more than 3. Consequently there is nothing in this sensitivity analyses that suggests that the West-Greenland population was able to sustain the same fishing

pressure as the Icelandic offshore population or even sustain the fishing pressure that developed during 1950s to the collapse of the West-Greenland population around 1970.

We also characterized the relationship between equilibrium F(5-12) and West Greenlandic offshore cod population productivity, i.e. the spawning stock biomass needed to produce one recruit (SBB/R) (Supplementary Figure S5).

Section S4 – Oceanographic model data

In order to describe recent historical ocean conditions we draw upon an archived ocean hindcast simulation conducted using the EC-Earth climate model configured in a decoupled, forced mode. The applied version (V2.2) of EC-Earth [4] is a fully coupled Atmosphere Ocean General Circulation Model (AOGCM), which builds on the Nucleus for European Modeling of the Ocean, NEMO system coupled to the LIM2 sea-ice module. The ocean configuration of NEMO has a resolution of 1°×1° with a meridional refinement to 1/3° at the equator, referred to as the ORCA1 grid. Here, the singularity at the North Pole is avoided by use of a tri-polar grid with poles over land (Siberia, Canada, Antarctica). Using 42 vertical z-layers, vertical ocean resolution increases from 10m at the surface to 300m at depth and reaches down to 5,500m. The large scale ocean circulation in the coupled system is in good agreement with the present views (see [5] and references herein) and general characteristics of the Arctic - subarctic ice-ocean exchange system has been convincingly assessed in [6]. The uncoupled simulation for the period 1948-2011 is forced by 6-hourly atmospheric NCEP reanalysis data [7]. Runoff is prescribed from climatology and we make use of sea surface salinity restoring (app. 180 days for a 10m mixed layer). Using an annually permuted NCEP forcing sequence [8], an independent 300 years spin-up has been performed and the quasi equilibrium climate state of the ocean simulations has shown a modest drift in water mass properties relative to climatology. We computed the mean, maximum and minimum annual sea surface temperatures, bottom temperatures and barotropic stream function to map dynamically connected regions at the model grid and interpolate to a 7x7 km resolution to refine the coastline. Furthermore**,** we extracted climatic information for each year and location of each fish

recorded and we performed the ordination to visualize climatic niche differences between the two main spawning groups: West Greenland offshore and Iceland offshore (see Supplementary Figure S2).

Section S4 – Habitat suitability modeling - Evaluating population level suitability with dispersal

We computed the genetic proportion of the Iceland offshore population relative to the West Greenland offshore in each NAFO divisions (1A - 1F) for each time period. For each division and each time period, we computed the least-cost distance from the center of each division to the southern tip of Greenland, accounting for climatic suitability either at the species level or specific to the Iceland offshore population using the gdistance package in R. We computed this distance from the southern tip of Greenland, because this area corresponds to the entry point of the Iceland offshore population into west Greenland waters (Figure 3b). We also computed a static sea distance from this point to each NAFO division. Northwest Atlantic Fisheries Organization (NAFO) members send their annual compilation of information on national catches and landings to the NAFO secretariat, and NAFO fisheries statistics have been compiled since 1951 [9].The database contains information on annual catches by species, subareas, country and year. We computed the average landing of cod in each division during the five years prior to the date with genetic sampling. We then related the proportion of Iceland offshore population in each NAFO division to distance between the center of the NAFO division to the southern tip of Greenland according to three distance metrics a) geographic distance “as the fish swims”, b) a least cost path through a habitat suitability surface defined for cod (all spawning populations combined), and c) a least cost path derived through a habitat suitability surface derived for each spawning population. We compared the explained variance of each predictor (R^2) and tested the significance using a Wald-z test.

Moreover, to visualize climatic niche differences between the two spawning groups, we performed a Principal Component Analysis (PCA) on the mixed-stock data. We extracted climatic information for each year and location of each otolith recorded and we performed the ordination to contrast the West Greenland offshore and Iceland offshore population niches. Results showed that the West Greenland offshore

population occured predominantly in colder sea surface temperatures, while the Iceland offshore population occupied a relatively broader range of temperature conditions including warmer temperatures.

Section S5 – Supplementary Figure S1 to S5

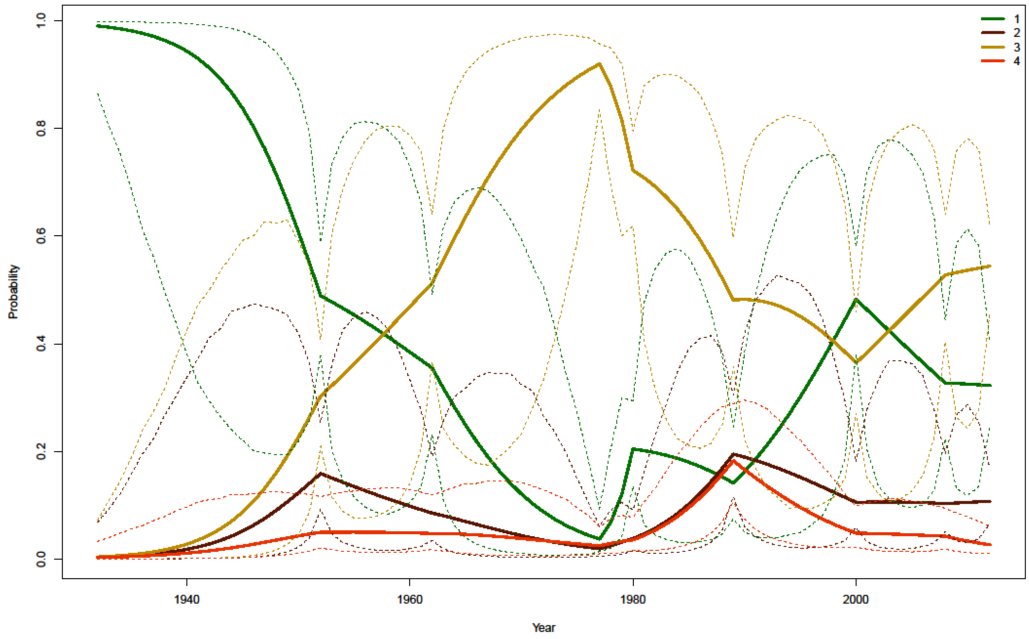

Supplementary Figure S1. Probability distributions and their 95% confidence intervals (dotted lines) for contribution of the four populations to the total catch across the studied time period. Each color represents one of the four spawning populations of cod: West Greenland offshore (green, 1), West Greenland inshore (brown, 2), Iceland offshore (dark yellow, 3) and Iceland inshore (red, 4).

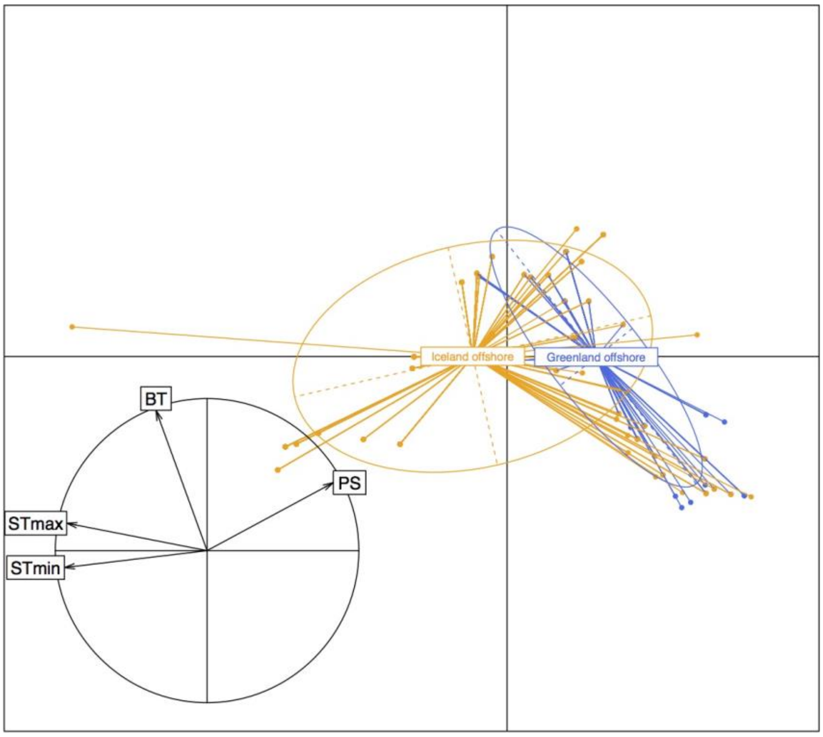

Supplementary Figure S2. Plot of a principal component analysis (PCA) on climatic information from coupling the fish occurrences and the Atmosphere Ocean Circulation Model (AOGCM) environmental layers (max and min sea surface temperature, STmax and STmin; bottom temperature, BT; barotropic stream function, PS). Component 1 is plotted on the X axis, while component 2 is on the Y axis. The West Greenland offshore (blue) and Iceland offshore (in orange) spawning populations are shown.

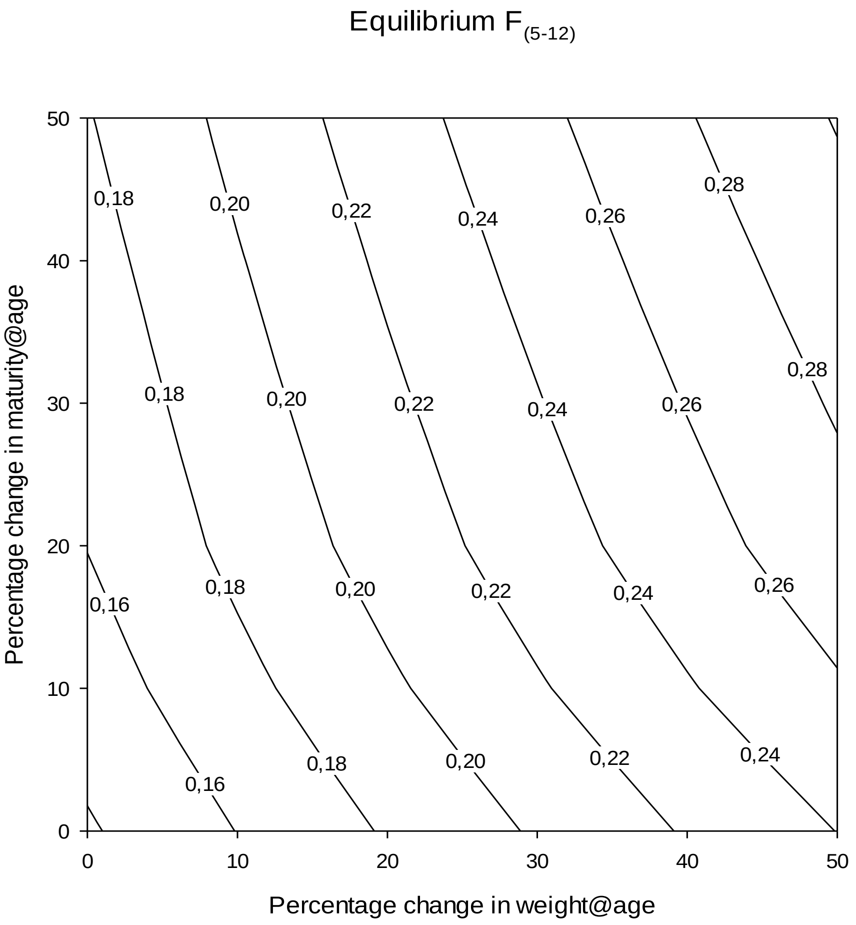

Supplementary Figure S3. Sensitivity analysis of the effect of increasing weight and maturity-at-age on the equilibrium fishing mortality.

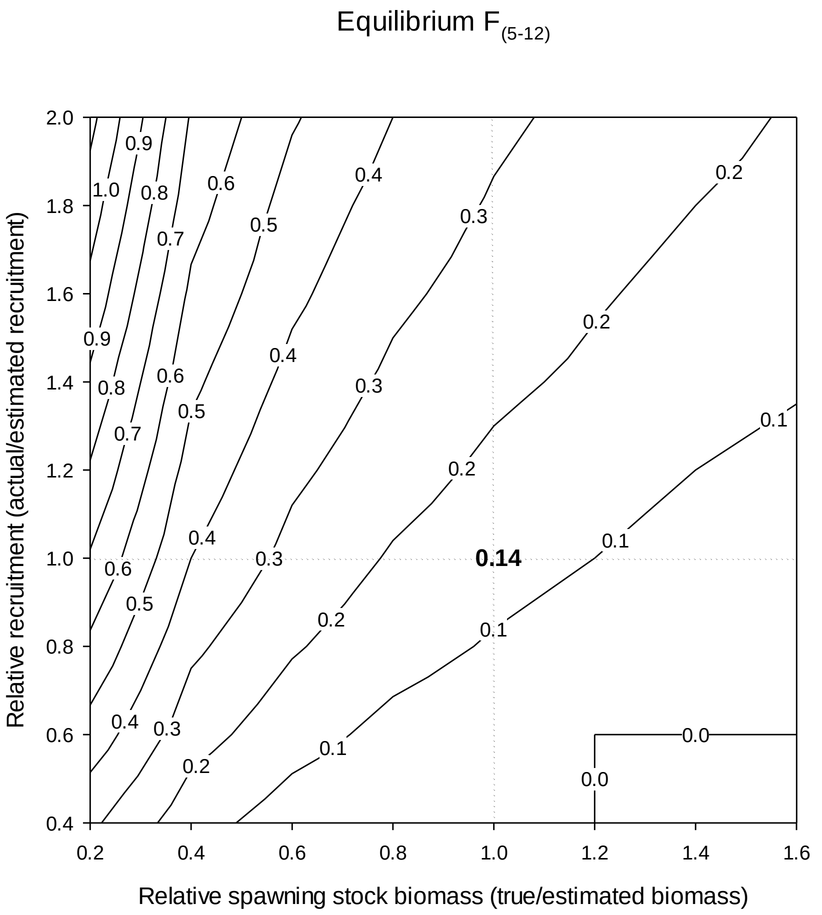

Supplementary Figure S4. Sensitivity analysis of the effect of errors in the spawning stock biomass (SSB) and recruitment estimation on equilibrium fishing mortality. A relative spawning stock biomass of 1.6 means that the true SSB was 60% larger than originally estimated. A relative recruitment of 2.0 means that recruitment is double what was originally estimated.

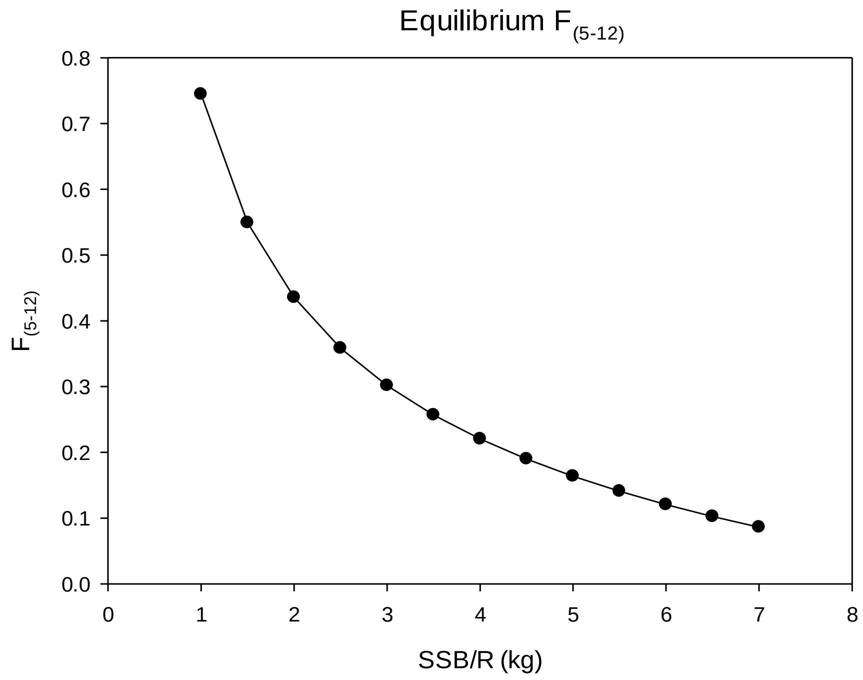

Supplementary Figure S5. The relationship between spawning stock productivity (biomass needed to produce one recruit) and equilibrium fishing mortality in the West Greenlandic offshore cod population.

Section S6 – Supplementary Tables S1 to S4

Supplementary Table S1. Archived tissue samples of Atlantic cod (*Gadus morhua*) collected between late June and January in 1932, 1952, 1962, 1977, 1980, 1989, 2000, 2008 and 2012 across the NAFO divisions in West Greenland.

**Year**	**NAFO division**					
	**1A**	**1B**	**1C**	**1D**	**1E**	**1F**
1932		26	33	31		
1952		10	21	22	17	20
1962		12	10	23	3	11
1977				36	28	37
1980		30		39	30	
1989				31	16	36
2000	6	42	30	26		8
2008		26		13	11	27
2012	18	35	26	29	25	28

Supplementary Table S2. Archived tissue samples of Atlantic cod (*Gadus morhua*) collected between late June and January in 1932, 1952, 1962, 1977, 1980, 1989, 2000, 2008 and 2012 across the NAFO divisions in West Greenland.

**1A**	**Sampling years**								
	**1932**	**1952**	**1962**	**1977**	**1980**	**1989**	**2000**	**2008**	**2012**
**June**									16
**July**									
**August**							7		
**September**									
**October**									
**November**									
**December**									
**January**									

**1B**	**Sampling years**								
	**1932**	**1952**	**1962**	**1977**	**1980**	**1989**	**2000**	**2008**	**2012**
**June**								10	25
**July**							37	13	
**August**	24	3	2						
**September**		3	1						

**October**		2	1						
**November**		1	2		26				
**December**			1						
**January**		1	1						
**1C**				**Sampling**	**years**				
	**1932**	**1952**	**1962**	**1977**	**1980**	**1989**	**2000**	**2008**	**2012**
**June**			2						1
**July**	20	15	2				28		21
**August**	13		1						
**September**		1	1						
**October**		1	1						
**November**			1						
**December**									
**January**			1						
**1D**				**Sampling**	**years**				
	**1932**	**1952**	**1962**	**1977**	**1980**	**1989**	**2000**	**2008**	**2012**
**June**		4	2						8
**July**		8				6	22	8	19

**August**	25	5	1			21			
**September**		2	3	17					
**October**		1	9	17	39				
**November**			1						
**December**									
**January**			1						
**1E**				**Sampling**	**years**				
	**1932**	**1952**	**1962**	**1977**	**1980**	**1989**	**2000**	**2008**	**2012**
**June**			3	27	29				
**July**		16							22
**August**		1			13			10	
**September**									
**October**									
**November**									
**December**									
**January**									
**1F**				**Sampling**	**years**				
	**1932**	**1952**	**1962**	**1977**	**1980**	**1989**	**2000**	**2008**	**2012**

**June**							
**July**		1					24
**August**			34	28		22	
**September**	19				8		
**October**	1						
**November**							
**December**		6					
**January**		1					

Supplemetary Table S3. SSB per recruit analysis based on input data from the West Greenland offshore cod population. Natural mortality is 0.2 and the VPA fishing mortalities are the average values from 1961-1963.

**Age (yr)**	**F from VPA**	**Equilibrium F**	**Z**	**Weight (kg)**	**Maturity ogive**	**Stock (n)**	**Catch (n)**	**Yield (kg)**	**SSB (kg)**
3	0.02	0.01	0.21	0.58	0.01	1.00	0.01	0.00	0.01
4	0.10	0.03	0.23	1.28	0.03	0.81	0.02	0.03	0.03
5	0.27	0.08	0.28	1.72	0.11	0.65	0.04	0.08	0.12
6	0.38	0.11	0.31	2.51	0.32	0.49	0.05	0.12	0.39
7	0.56	0.16	0.36	3.52	0.61	0.36	0.05	0.17	0.77
8	0.55	0.16	0.36	4.66	0.83	0.25	0.03	0.15	0.97
9	0.48	0.14	0.34	5.07	0.94	0.18	0.02	0.10	0.84
10	0.48	0.14	0.34	5.68	0.98	0.13	0.01	0.08	0.70
11	0.52	0.15	0.35	5.37	0.99	0.09	0.01	0.06	0.48
12	0.62	0.18	0.38	8.65	1.00	0.06	0.01	0.08	0.55
13	0.60	0.17	0.37	9.58	1.00	0.04	0.01	0.06	0.42
14	0.48	0.14	0.34	9.60	1.00	0.03	0.00	0.03	0.29
	F(5-12):	*0.14*					*Sum:*	*0.97*	*5.57*

Supplementary Table S4. SSB per recruit analysis based on input data from the Icelandic offshore cod population. Natural mortality is 0.2 and the VPA fishing mortalities are the average values from 1961-1963. All other input data are averages from 1955-2005.

**Age (yr)**	**F from VPA**	**Equilibrium F**	**Z**	**Weight (kg)**	**Maturity ogive**	**Stock (n)**	**Catch (n)**	**Yield (kg)**	**SSB (kg)**
3	0.07	0.09	0.29	0.64	0.01	1.00	0.15	0.10	0.01
4	0.23	0.29	0.49	1.37	0.04	0.75	0.21	0.29	0.04
5	0.37	0.48	0.68	2.19	0.13	0.46	0.13	0.28	0.11
6	0.49	0.63	0.63	3.25	0.36	0.23	0.06	0.20	0.22
7	0.60	0.77	0.97	4.41	0.65	0.10	0.04	0.17	0.27
8	0.70	0.90	1.10	6.18	0.79	0.04	0.02	0.13	0.21
9	0.74	0.95	1.15	7.30	0.81	0.01	0.01	0.06	0.10
10	0.76	0.98	1.18	8.51	0.98	0.00	0.00	0.03	0.05
11	0.74	0.96	1.16	9.81	1.00	0.00	0.00	0.01	0.02
12	0.67	0.87	1.07	11.57	0.99	0.00	0.00	0.00	0.01
13	0.53	0.68	0.88	13.17	1.00	0.00	0.00	0.00	0.00
14	0.53	0.68	0.88	15.98	1.00	0.00	0.00	0.00	0.00
	F(5-12):	*0.82*					*Sum:*	*1.39*	*1.06*

References

1. Therkildsen, N.O. *et al*. Spatiotemporal SNP analysis reveals pronounced biocomplexity at the northern range margin of Atlantic cod *Gadus morhua*. *Evolutionary Applications* **6**, 690–705 (2013).
2. ICES. *Report of the North-Western Working Group (NWWG), 1 – 8 May 1996*. ICES ICES Document CM 1996/Assess: 15. *Scientific report* (1996).
3. Hovgård, H. & Wieland, K. Fishery and environmental aspects relevant for the emergence and decline of Atlantic cod (Gadus morhua) in West Greenland waters. Resiliency of gadid stocks to fishing and climate change. Alaska Sea Grant, University of Alaska Fairbanks (2008).
4. Hazeleger, W. *et al*. EC-Earth V2. 2: description and validation of a new seamless earth system prediction model. *Climate dynamics* **39**, 2611-2629 (2012).
5. Sterl, A. *et al*. A look at the ocean in the EC-Earth climate model. *Climate dynamics* **39**, 2631-2657 (2012).
6. Koenigk, T. *et al*. Arctic climate change in 21st century CMIP5 simulations with EC-Earth. Climate dynamics, 40(11-12), 2719-2743 (2013).
7. Kalnay, E. *et al*. The NCEP/NCAR 40-year reanalysis project. *Bulletin of the American meteorological Society* **77**, 437-471 (1996).
8. Olsen, S.M. & Schmith, T. North Atlantic–Arctic Mediterranean exchanges in an ensemble hindcast experiment. *Journal of Geophysical Research: Oceans (1978–2012)* **112**, (2007).
9. WEBSITE: http:\\www.nafo.int/data/rames/data.html
